# Alternative evaluation methods in medical education

**DOI:** 10.3205/zma001739

**Published:** 2025-02-17

**Authors:** Marjo Wijnen-Meijer

**Affiliations:** 1 TUD Dresden University of Technology, Medical Faculty and University Hospital Carl Gustav Carus, Institute of Medical Education, Dresden, Germany

## Editorial

Quality assurance and the research and development of teaching are legally required and central components of higher education didactics. This is particularly true in medical education, which is shaped by specific demands and practice-oriented learning processes. 

Feedback plays a crucial role in systematically capturing teaching quality, optimizing learning processes, and supporting both teachers and students in their development [1]. Two main types of feedback can be distinguished: feedback to teachers, which reflects their teaching quality and identifies areas for improvement, and feedback to students, which evaluates their performance and progress.

This article focuses on feedback to teachers, which is essential for the development of teaching and the maintenance of teaching quality.

Traditionally, universities use standardized student evaluations (SETs) in the form of standardized questionnaires, which are widely used to assess the quality of courses and teaching performance because of their simple handling and comparability [2]. However, these approaches often capture only snapshots and are limited in significance. In the medical context, where clinical exercises, simulations, and interprofessional learning play a central role, such methods often fail to adequately capture the complex teaching and learning processes. Numerous studies have shown that SETs are often strongly influenced by factors such as student expectations, gender, or ethnicity, and therefore may not always provide an objective assessment of teaching quality [3], [4]. At the same time, external factors such as course size or exam stress can influence the results [5], making them more of a “satisfaction analysis” rather than a true teaching evaluation [6].

In this context, the development and implementation of alternative evaluation approaches is becoming increasingly important. Methods such as realistic evaluation, as well as focus groups or peer feedback, allow for a more differentiated view of the contexts, dynamics, and effects in medical teaching situations. 

The realistic evaluation, developed by Pawson and Tilley, focuses on understanding how programs work in specific contexts and the mechanisms that trigger these effects [7]. This method is particularly useful in medical education, as it not only examines outcomes but also the underlying conditions and processes. It is based on the principle: “What works, for whom, in what circumstances, and how?”. In teaching, this approach could represent a shift from purely summative assessments to analyzing the underlying learning processes and contexts. For example, one could investigate why a particular simulation training is successful for some students but not for others. The method requires both qualitative and quantitative data, such as interviews, observations, or performance assessments. Therefore, it is especially useful for studying complex teaching settings, as they occur in medical education.

In this context, realistic evaluation can be applied to analyze the effects of training programs, teaching methods or curriculum changes. The underlying core principles include mechanisms, contexts, and outcomes.

Mechanisms refer to the processes or underlying factors that explain how an intervention works. In a medical simulation, for instance, the mechanism might be the opportunity for learners to practice decision-making in a realistic environment.

Contexts involve the conditions or environments in which the intervention takes place. For example, learner engagement might depend on the availability of instructors or the cultural norms within the institution.

Finally, outcomes are the results or changes of the intervention. These might include improved clinical skills, enhanced teamwork, or increased self-confidence among participating students.

Insights from realistic evaluations help prepare instructional interventions to different types of learners and underlying contexts. In medical education, this could mean adapting teaching strategies for different groups, such as international students, or adjusting clinical instruction to the available resources at a hospital.

Peer observation can also be a valuable source of feedback, highlighting strengths and areas for development in teaching [8]. This can be done using an observation guide, although it is not always necessary. The key is to clearly define the observational focus in advance.

Furthermore, focus groups can be a valuable addition to other evaluation methods in medical education, such as student evaluations. They provide an opportunity to collect qualitative student feedback and offer a more holistic perspective on teaching quality. However, the main disadvantage is that focus groups are time-consuming and require facilitation experience to ensure productive discussions [9]. Despite these challenges, focus groups can be a valuable tool for assessing and improving medical education, as they provide detailed, qualitative insights, encourage communication between students and instructors, and can contribute to more effective, student-centered teaching design [10].

Finally, student learning outcomes are also a useful feedback source for instructors [11]. This can be achieved through the analysis of exam results in the traditional sense, but also by using quiz questions during lectures to gain a current understanding of what students have comprehended and where knowledge gaps still persist.

In this issue, various didactic interventions are evaluated. The article by Papan et al. describes the effect of an elective course on antimicrobial stewardship (AMS) [12]. The article by Ruck et al. evaluated the long-term effect of a smoking cessation counselling course using online questionnaires [13]. Tom Dreyer and colleagues compared the effect of two different teaching formats based on examination results [14]. Junga et al. compare the evaluations of compulsory and elective clerkships [15]. Brinkmann et al. have evaluated Mind-Body Medicine courses at two different universities using questionnaires and focus groups [16]. And Hofhansi et al. describe students’ experiences with supervision, which were collected using questionnaires [17].

Enjoy reading this interesting issue!

## Author’s ORCID

Marjo Wijnen-Meijer: [0000-0001-8401-5047]

## Competing interests

The author declares that she has no competing interests.
